# Supplementary material for: Autoantibodies to IL-1Ra and PGRN in severe COVID-19 are associated with inflammation-induced hyperphosphorylated antigen isoforms
Source: Nat Commun. 2026 May 27;17:4768. doi: 10.1038/s41467-026-73316-5 (PMC13219524; doi:10.1038/s41467-026-73316-5)
Supplement: Supplementary file 1 — Supplementary Information [file 41467_2026_73316_MOESM1_ESM.pdf]

Supplementary Information

Autoantibodies to IL-1Ra and PGRN in severe COVID-19 are associated with inflammation-induced hyperphosphorylated antigen isoforms

Lorenz Thurner[1#], Natalie Fadle[1], Bernhard Thurner[2], Igor Age Kos [1], Moritz Bewarder [1,3], Evi Regitz [1], Birgit Bette [1], Yvan Fischer [4], Vadim Lesan [1], Torben Rixecker [5], Marie-Christin Hoffmann[1,6], Klaus-Dieter Preuss [1], Claudia Schormann[1], Dominic Kaddu-Mulindwa[1,7], Klaus Roemer [1], Onur Cetin [1], Sebastian Mang [5,8], André Becker [5], Frederik Seiler [5], Christian Herr [5], Christian Lensch [5], Johannes Lehmann [9], Angela Thiel-Bodenstaff [9,10], Andreas Link [11], Christian Werner [11], Patrick Wuchter[12], Sixten Körper [13],Thorsten Pfuhl[14], Stefan Lohse [14], Jürgen Rissland[14], Katrin Thieser[14], Jan Pilch [15], Cihan Papan [16], Sophie Roth [16], J. Janne Vehreschild [17], Margarete Scherer[17], Isabel Bröhl [17], Patricia Wagner [17], Martin Witzenrath [18], Charlotte Thibeault [18], Ira an Haack [18], Lazar Mitrov [19], Sina M. Pütz [19], Jens-Peter Reese [20,21], Michael Krawczak[22], Eckard Hamelmann[23], Verena Kopfnagel[24], Karin Fiedler[17], Ramsia Geisler[17], Heike Valentin[25], Dana Stahl[25], Sabine Hanß[26], Sabine Ameling[27], Uwe Völker[27], Stefan Hansch[28], Marcus Dörr[29], Sabine Blaschke[30], Josephine Braunsteiner[31], Edgar Dahl[32], Daniel Pape[33], Astrid Petersmann[34], Stephan Stilgenbauer[35], Frank Bloos[36], Hubert Schrezenmeier[13], Frank Langer[37], Gereon Gäbelein[38], Bettina Friesenhahn-Ochs[9], Jochen Pfeifer[39], Michael Bauer[36], Sören Leif Becker[16], Frank Neumann[1], Michael Böhm[11], Gabriele Anton[40], Carsten Kuenne[41], Soni Savai Pullamsetti[41], Mario Looso[41], Robert Bals[5], Sigrun Smola[14], Patrick Meybohm[42], Marcin Krawczyk*[9,43,44], Philipp M. Lepper*[5,10], Christoph Kessel*[45]

*These authors jointly supervised this work

[1] José Carreras Center for Immuno- and Gene Therapy and Department of Internal Medicine I, Saarland University, Homburg/Saar, Germany

[2] Clinic Network Allgäu, Medical Care Center "Die Kindersprechstunde" Mindelheim, Mindelheim, Germany

[3] Department of Hematoly and Oncology, Bethanien Hospital, Frankfurt a.M., Germany

[4] Institute of Physiology, Medical Faculty, RWTH Aachen, Aachen, Germany

[5] Department of Internal Medicine V - Pulmonology, Allergology and Critical Care Medicine, Saarland University, Homburg, Germany
[6] Department of Pediatrics, University Hospital Erlangen, Erlangen, Germany
[7] Department of Oncology, Centre Hospitalier du Nord, Ettelbrück, Luxembourg
[8] Department of Intensive Care Medicine, UKE University Hospital Hamburg, Germany
[9] Department of Medicine II, Saarland University, Homburg, Germany
[10] Department of Emergency Medicine, Saarland University, Homburg, Germany
[11] Department of Internal Medicine III – Cardiology, Saarland University, Homburg, Germany
[12] Institute of Transfusion Medicine and Immunology, Heidelberg University, Medical Faculty Mannheim, German Red Cross Blood Service of Baden-Württemberg - Hessen gGmbH, Mannheim, Germany
[13] Institute of Clinical Transfusion Medicine and Immunogenetics Ulm, German Red Cross Blood andvTransfusion Service, Baden Wuerttemberg-Hessen, and University Hospital Ulm, Ulm, Germany
[14] Institute of Virology, University of Saarland, Homburg, Germany
[15] Institute of Clinical Haemostaseology and Transfusion Medicine, Homburg, Germany
[16] Center of Infectious disease, Institute of Medical Microbiology and Hygiene, University of Saarland, Homburg, Germany
[17] Institute for Digital Medicine and Clinical Data Sciences, Faculty of Medicine, Goethe University Frankfurt a. M., Germany
[18] Charité - Universitätsmedizin Berlin, Corporate Member of Freie Universität Berlin and Humboldt-Universität zu Berlin, Germany
[19] Department I of Internal Medicine, Center for Integrated Oncology (CIO) Aachen Bonn Cologne Duesseldorf, University of Cologne, Medical Faculty and University Hospital Cologne, Cologne, Germany
[20] Institute for Clinical Epidemiology and Biometry, University of Würzburg, Würzburg, Germany
[21] Faculty of Health, Technische Hochschule Mittelhessen (THM), Gießen, Germany
[22] Institute of Medical Informatics and Statistics, University Medical Center Schleswig-Holstein, Kiel University, Kiel, Germany
[23] Department of Pediatrics, Children's Center Bethel, Bielefeld, University Hospital OWL, University of Bielefeld, Bielefeld, Germany
[24] Hannover Unified Biobank, Hannover Medical School, Hannover, Germany
[25] Independent Trusted Third Party of the University Medicine Greifswald, Greifswald, Germany
[26] Department of Medical Informatics, University Medical Center Göttingen, Göttingen, Germany

[27] Department of Functional Genomics, Interfaculty Institute for Genetics and Functional Genomics, University Medicine Greifswald, Greifswald, Germany
[28] Department for Infectious Diseases and Infection Control, University Hospital Regensburg, Regensburg, Germany
[29] Department of Internal Medicine B, University Medicine Greifswald, Greifswald, Germany
[30] Emergency Department, University Medical Center Goettingen
[31] Center for Anesthesiology and Intensive Care Medicine, University Hospital UKE, Hamburg-Eppendorf, Hamburg, Germany
[32] RWTH centralized Biomaterial Bank (RWTH cBMB), Institute of Pathology, Medical Faculty, RWTH Aachen University, Aachen, Germany
[33] Department I of Internal Medicine, University Medical Center Schleswig-Holstein Campus Kiel, Kiel, Germany
[34] Institute for Clinical Chemistry and Laboratory Medicine, University Medicine Oldenburg, Oldenburg, Germany
[35] Comprehensive Cancer Center Ulm and Division of CLL-Internal Medicine III, Ulm University, Ulm, Germany
[36] Department of Anesthesiology and Intensive Care Medicine, Jena University Hospital, Jena, Germany
[37] Department of Thoracic Surgery, Saarland University Medical Center, Homburg/Saar, Germany
[38] Department of General, Visceral, Vascular and Pediatric Surgery, University of Saarland, Homburg, Germany
[39] Department of Pediatric Cardiology, University of Saarland, Saarland, Homburg, Germany Department
[40] Medical School East Westphalia-Lippe, Bielefeld University, Bielefeld, Germany
[41] Max-Planck Institute for Heart and Lung Research, Bad Nauheim, Germany
[42] Department of Anesthesiology, Intensive Care, Emergency and Pain Medicine, University Hospital Würzburg, Würzburg, Germany
[43] Department of Gastroenterology, Hepatology and Transplant Medicine, Medical Faculty, University of Duisburg-Essen, Essen, Germany
[44] Laboratory of Metabolic Liver Diseases, Centre for Preclinical Research, Department of General, Transplant and Liver Surgery, Medical University of Warsaw, Warsaw, Poland
[45] Department of Pediatric Rheumatology and Immunology, University of Münster, Münster, Germany

[#]Corresponding author:
Lorenz Thurner, MD

Dept. of Internal Medicine I and José-Carreras-Center for Immuno- and Gene Therapy
Saarland University Medical School
D-66421 Homburg/Saar, Germany
e-mail: lorenz.thurner@uks.eu;
Phone: +49-6841-1615362

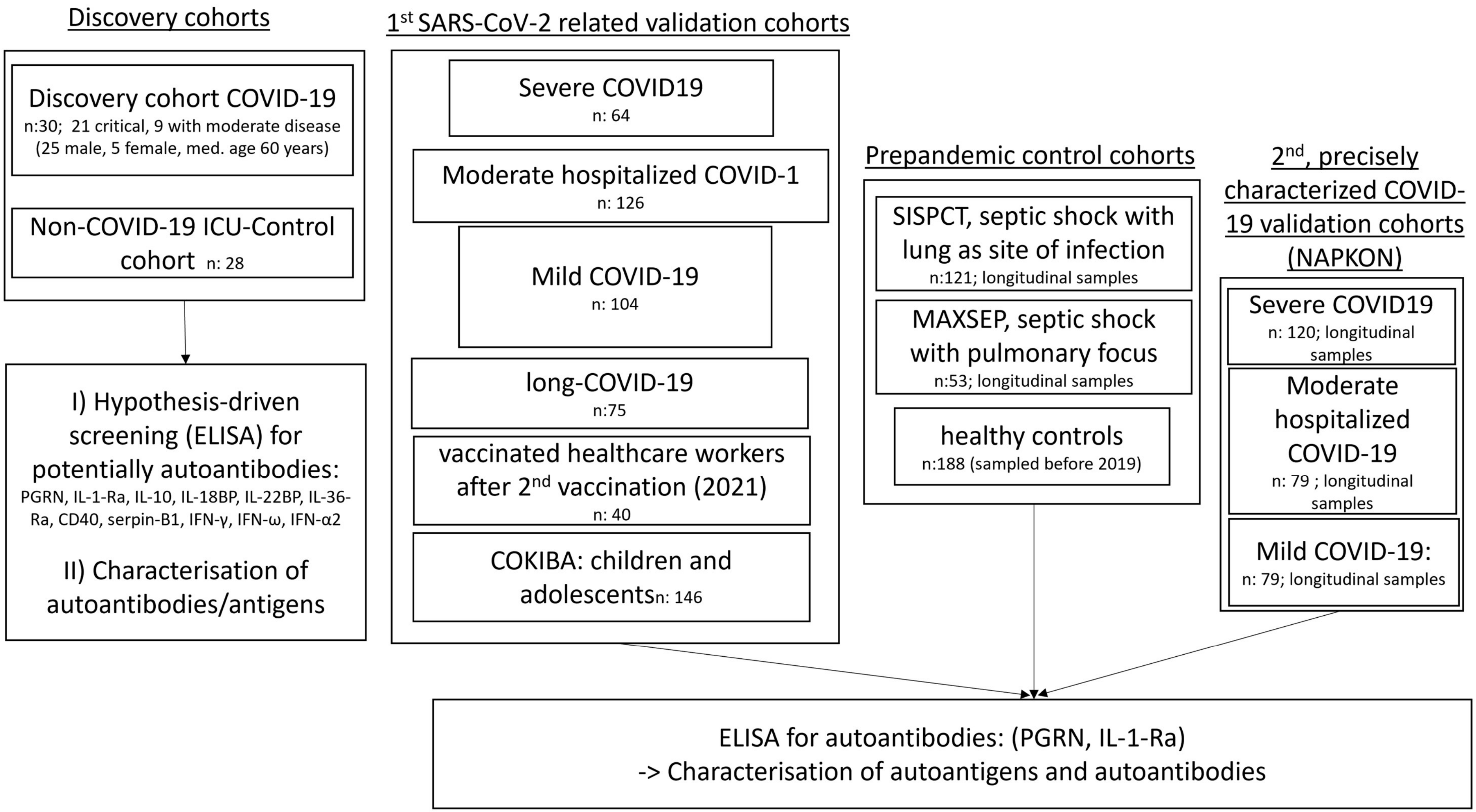

Supplementary Figure 1. Study cohorts and linked analysis.

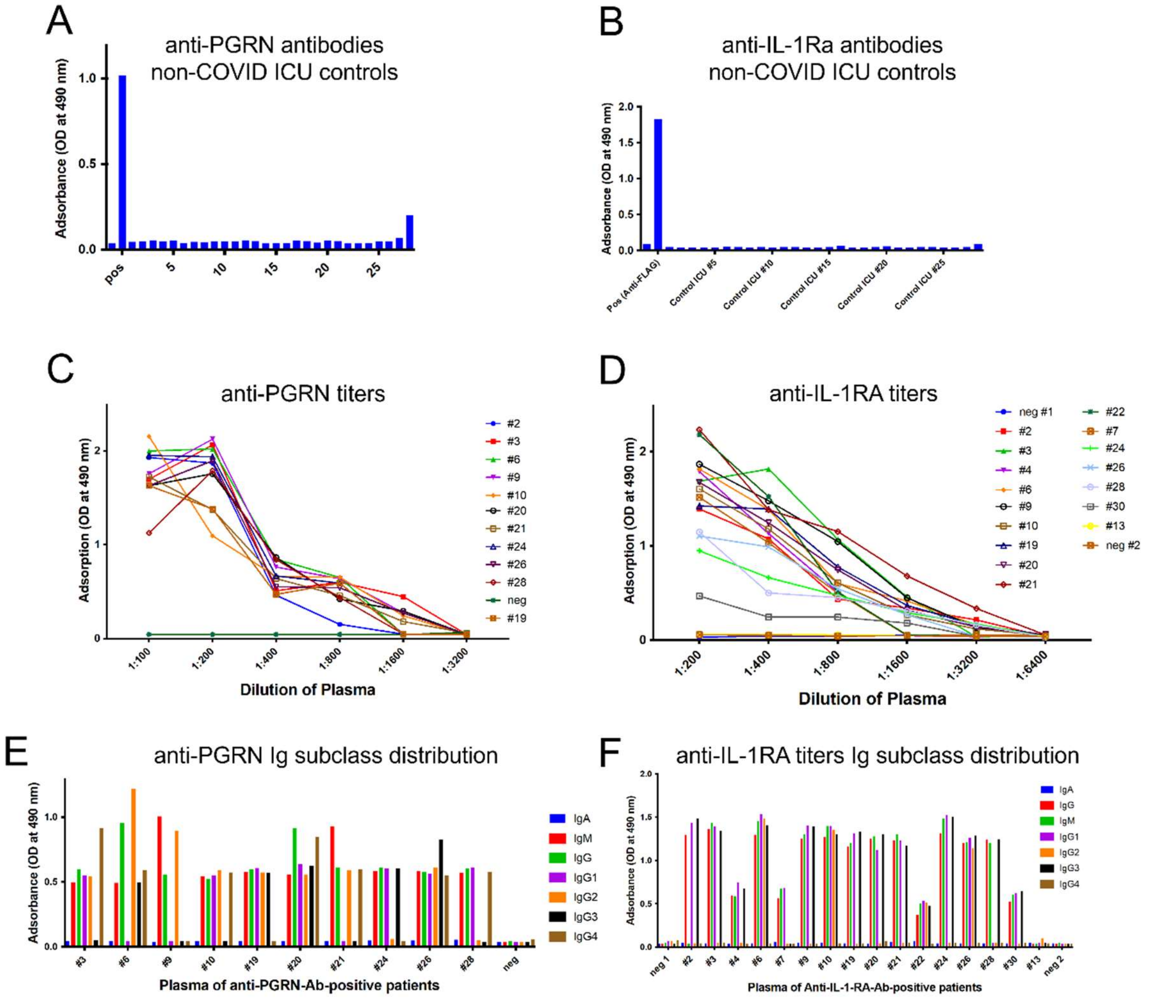

Supplementary Figure 2. Anti-PGRN and anti-IL-1Ra antibodies in discovery cohort ICU controls as well as autoantibody titers and immunoglobulin (Ig) subclass distribution among seropositive discovery cohort COVID19 patients. (**A**, **B**) Anti-PGRN (**A**) and anti-IL-1Ra IgG levels (**B**) in plasma samples of non-COVID19 ICU patients (n=28) were assessed by in-house ELISA. (**C**, **D**) Titers of anti-PGRN (**C**) and anti-IL-1Ra antibodies (**D**) were determined by serial dilution of plasma previously tested as seropositive (**Fig. 1A**) using respective in-house ELISAs. (**E**, **F**) Ig subclass distribution of anti-PGRN (**E**) and anti-IL-1Ra antibodies (**F**) in plasma previously tested as seropositive (**Fig. 1A**) was determined by using Ig-subclass specific secondary antibodies in respective in-house anti-PGRN and anti-IL-1Ra ELISA platforms.

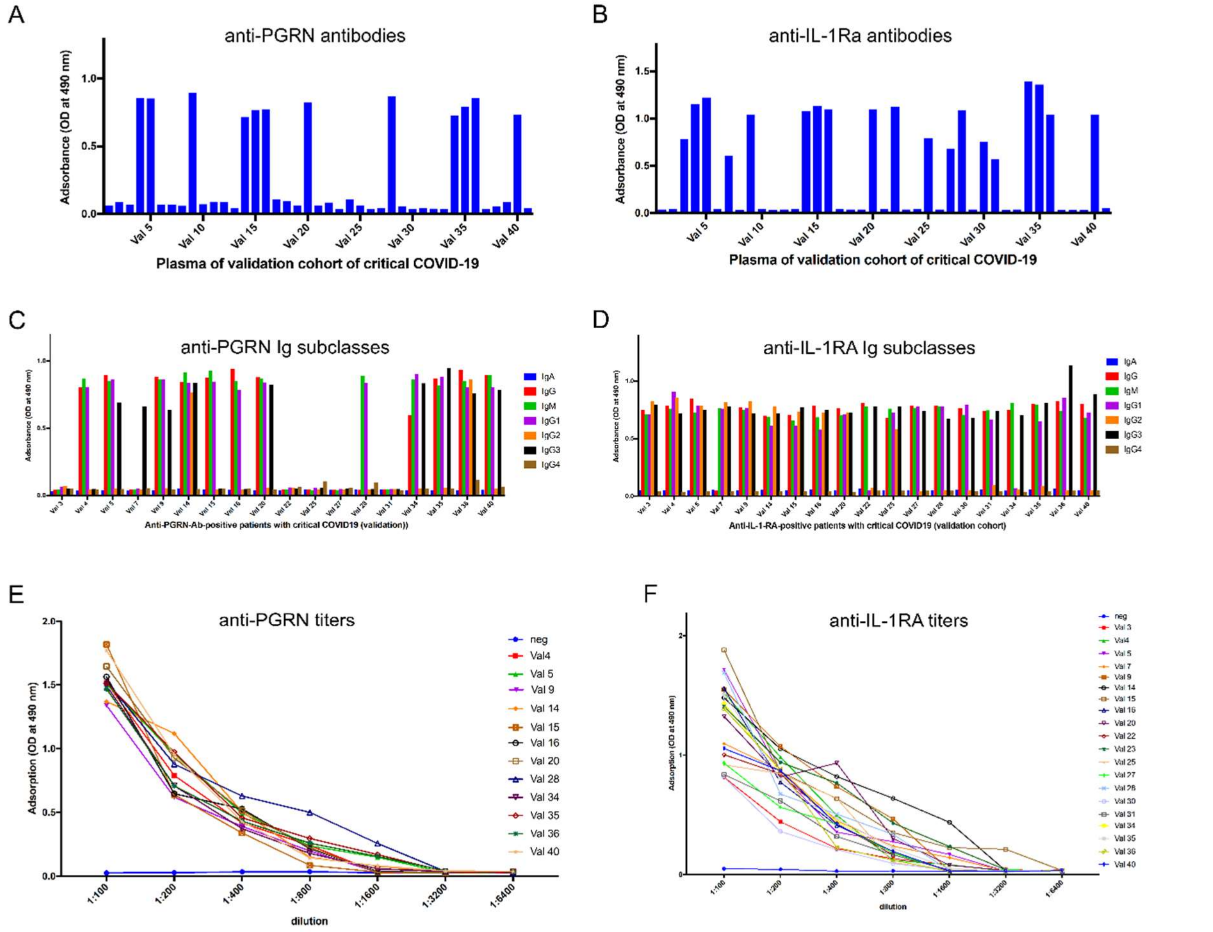

Supplementary Figure 3. Exemplary anti-PGRN and anti-IL-1Ra antibodies titers and immunoglobulin (Ig) subclass distribution among validation cohort 1 COVID19 patients. (**A**, **B**) Exemplary anti-PGRN (**A**) and anti-IL-1Ra IgG ELISA data (**B**) of plasma samples obtained from sever/critical COVID19 patients enrolled in validation cohort 1 (n=41). (**C**, **D**) Ig subclass distribution of anti-PGRN (**C**) and anti-IL-1Ra antibodies (**D**) in validation cohort plasma was determined by using Ig-subclass specific secondary antibodies in respective in-house anti-PGRN and anti-IL-1Ra ELISA platforms. (**E**, **F**) Titers of anti-PGRN (**E**) and anti-IL-1Ra antibodies (**F**) were determined by serial dilution of plasma previously tested as seropositive using respective in-house ELISAs.

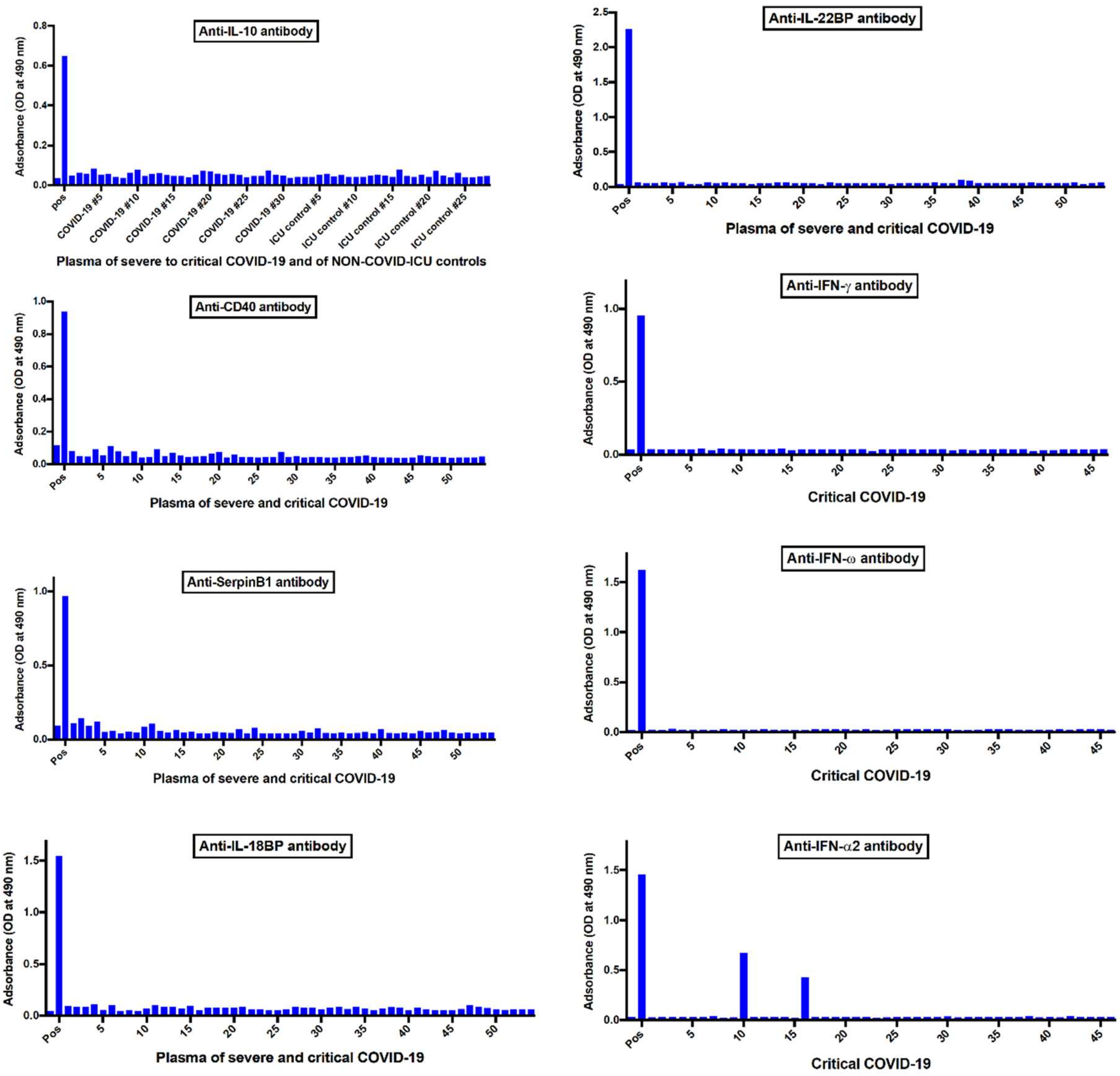

Supplementary Figure 4. Screening of severe and critical COVID19 patients' plasma samples of varying numbers for antibody reactivities with possible antigens with reported anti-inflammatory role (CD40, IL-10, IL-18bp, IL-22bp, IL-36Ra and serpin-B1) or relevance in COVID19 (IFNα2, IFNω, IFNγ). For screening of plasma samples, N-terminally FLAG-tagged antigens were expressed in HEK293 cells and immobilized on ELISA plates via anti-FLAG antibody.

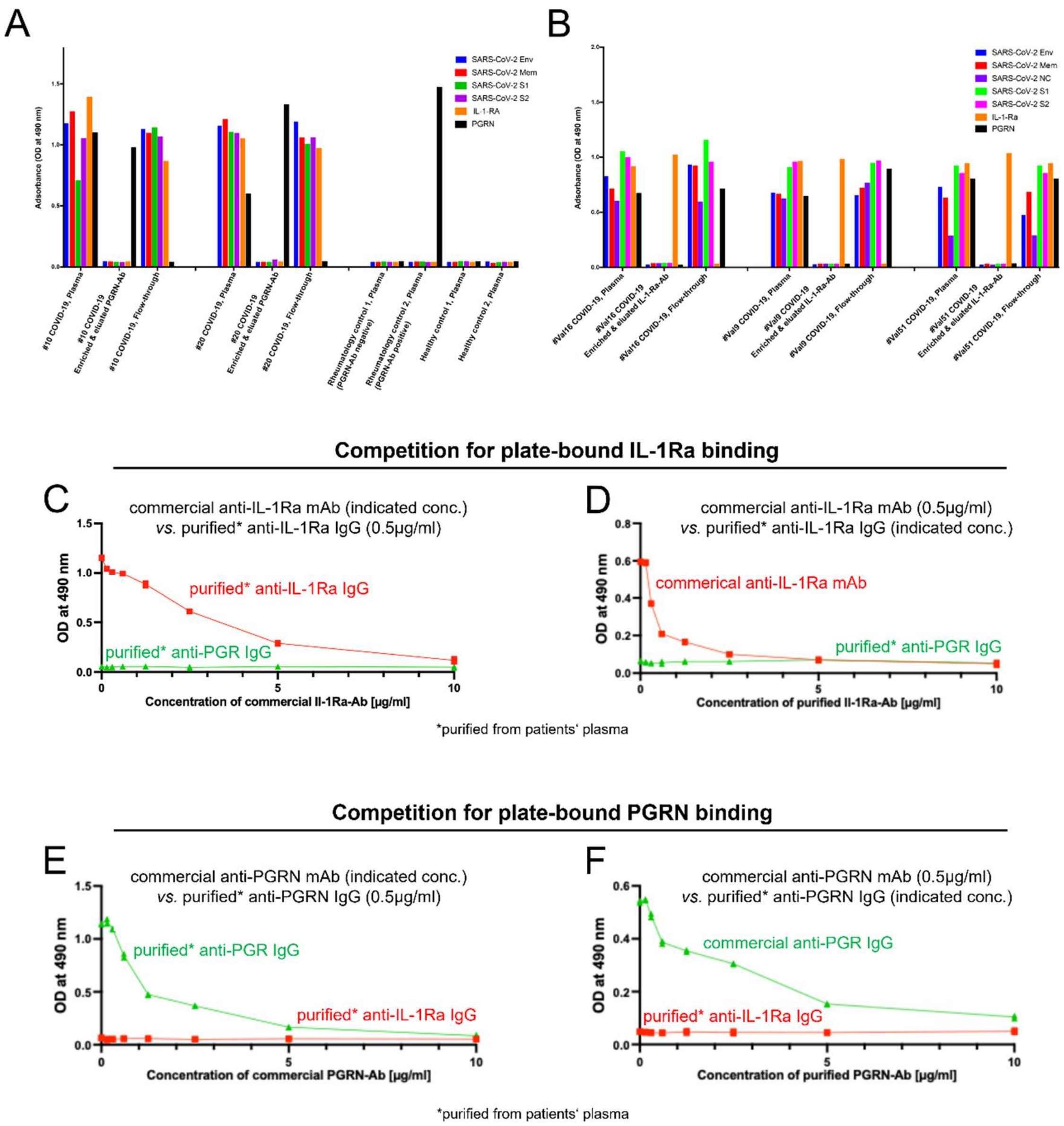

Supplementary Figure 5. Test for crossreactivity of anti-PGRN and anti-IL-1Ra IgG in patients' plasma with structural SARS-CoV-2 proteins and competition with commercial antibodies. (**A**, **B**) Anti-PGRN (**A**) and anti-IL-1Ra antibodies (**B**) were enriched from plasma previously tested seropositive was eluted PGRN-. Enriched anti-PGRN (**A**) and anti-IL-1Ra antibodies (**B**) as well as respective flow-through from column purification was tested for IgG cross-reactivity with recombinant HIS-tagged SARS-CoV-2 S1-, S2-, E- or M-protein, as well as recombinant FLAG-tagged human IL-1RA (anti-PGRN antibodies) or PGRN (anti-IL-1Ra antibodies) by ELISA. (**C**, **D**) Competition ELISAs on plate-bound rhIL-1Ra of commercial murine anti-hIL-1Ra mAb (at indicated concentrations (**C**) or 0.5µg/ml (**D**)) *vs.* anti-IL-1Ra IgG purified from patient's plasma (at 0.5µg/ml (**C**) or indicated concentration (**D**)). (**E**, **F**) Competition ELISAs on plate-bound rhPGRN of commercial murine anti-hPGRN mAb (at indicated concentrations (**C**) or 0.5µg/ml (**D**)) *vs.* anti-PGRN IgG purified from patient's plasma (at 0.5µg/ml (**C**) or indicated concentration (**D**)).

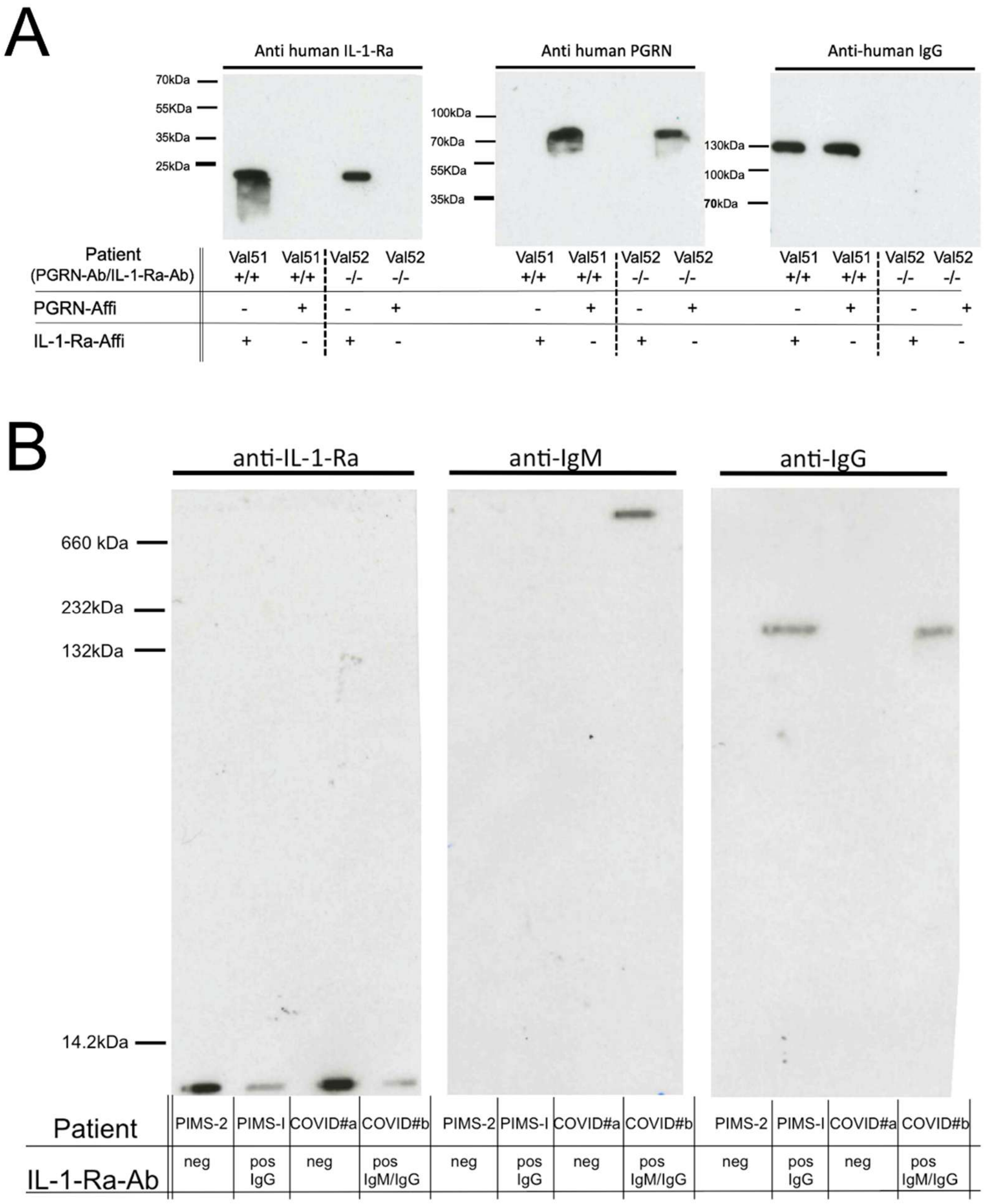

Supplementary Figure 6. Immune complexing of purified or endogenous antigen by anti-PGRN and anti-IL-1Ra antibodies in validation cohort patients' plasma. (**A**) IL-1Ra and PGRN were purified by goat or mouse antibodies coupled to Affi-gel HZ from plasma of critical COVID19 patients previously tested positive (Val51) or negative (Val52) for anti-PGRN and anti-IL-1-Ra-antibodies. IgG immune complexes with purified IL-1-Ra and PGRN were by western blot. (**B**) Western blot of SDS containing gradient gels using non-reducing sample preparation conditions of immunoprecipitated IL-1-Ra purified from plasma of patients with PIMS/MIS-C or adult patients with COVID19, previously tested seropositive or negative for anti-IL-1Ra antibodies. Western blots were stained with anti-IL-1Ra, anti-IgM or anti-IgG detection antibodies.

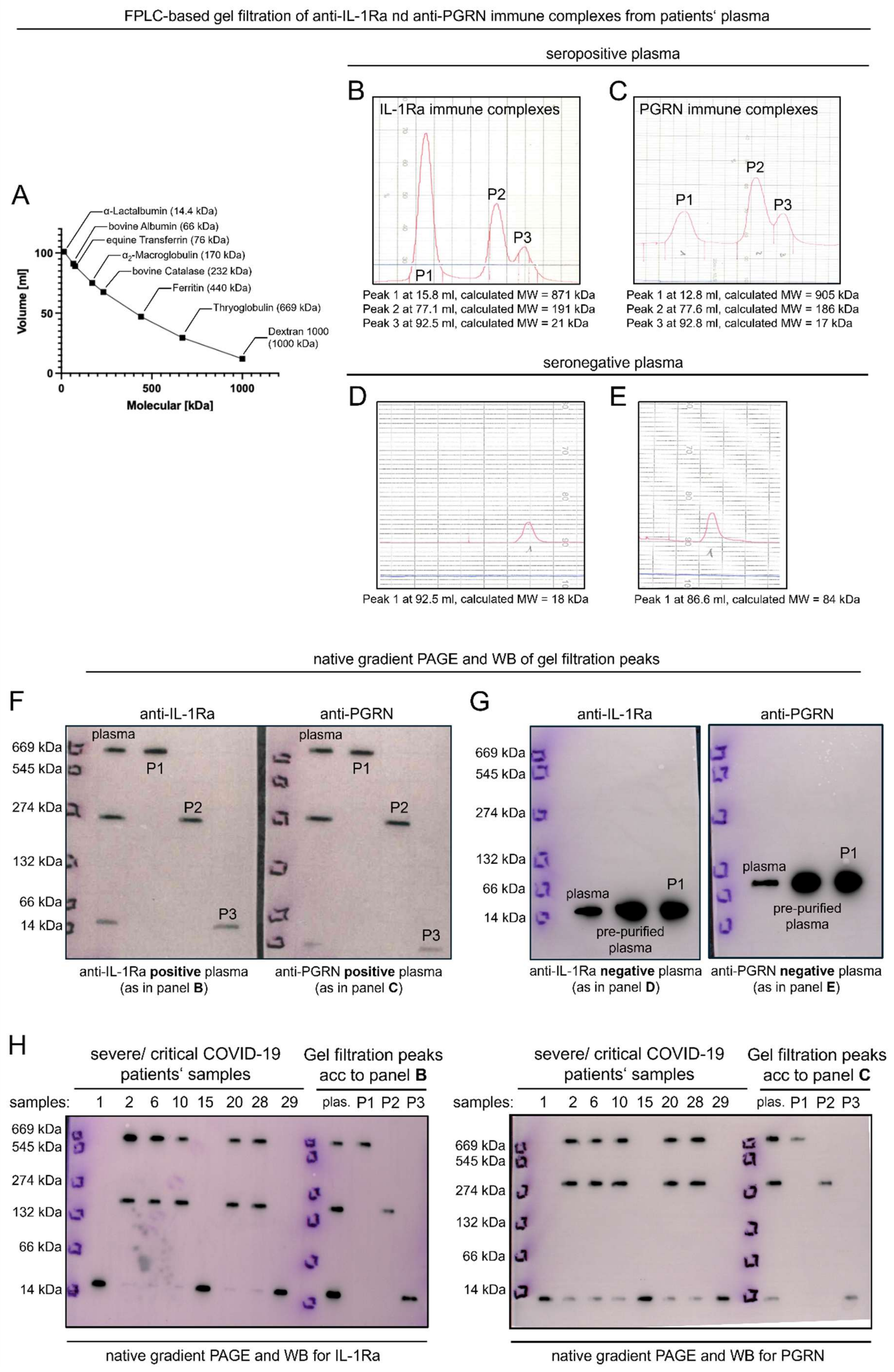
FPLC-based gel filtration of anti-IL-1Ra nd anti-PGRN immune complexes from patients‘ plasma
A
α-Lactalbumin (14.4 kDa)
bovine Albumin (66 kDa)
equine Transferrin (76 kDa)
α2-Macroglobulin (170 kDa)
bovine Catalase (232 kDa)
Ferritin (440 kDa)
Thryoglobulin (669 kDa)
Dextran 1000 (1000 kDa)
Volume [ml]
Molecular [kDa]
seropositive plasma
B
IL-1Ra immune complexes
P1
P2
P3
Peak 1 at 15.8 ml, calculated MW = 871 kDa
Peak 2 at 77.1 ml, calculated MW = 191 kDa
Peak 3 at 92.5 ml, calculated MW = 21 kDa
C
PGRN immune complexes
P1
P2
P3
Peak 1 at 12.8 ml, calculated MW = 905 kDa
Peak 2 at 77.6 ml, calculated MW = 186 kDa
Peak 3 at 92.8 ml, calculated MW = 17 kDa
seronegative plasma
D
Peak 1 at 92.5 ml, calculated MW = 18 kDa
E
Peak 1 at 86.6 ml, calculated MW = 84 kDa
native gradient PAGE and WB of gel filtration peaks
F
anti-IL-1Ra
anti-PGRN
669 kDa
545 kDa
274 kDa
132 kDa
66 kDa
14 kDa
plasma
P1
P2
P3
anti-IL-1Ra positive plasma (as in panel B)
anti-PGRN positive plasma (as in panel C)
G
anti-IL-1Ra
anti-PGRN
plasma
pre-purified plasma
P1
anti-IL-1Ra negative plasma (as in panel D)
anti-PGRN negative plasma (as in panel E)
H
severe/ critical COVID-19 patients‘ samples
Gel filtration peaks acc to panel B
Gel filtration peaks acc to panel C
samples: 1 2 6 10 15 20 28 29 plas. P1 P2 P3
native gradient PAGE and WB for IL-1Ra
native gradient PAGE and WB for PGRN

Supplementary Figure 7. Characterization of IL-1Ra and PGRN immune complexes. (**A**-**E**) FPLC gel filtration of free and antibody-bound IL-1Ra and PGRN. (**A**) Calibration curve of the Superdex 200 filtration column. (**B**-**E**) Affi-Matrix pre-purified plasma samples, either IL-1Ra-Ab pos/PGRN-Ab neg or IL-1Ra-Ab neg/PGRN-Ab pos or seronegative for both anti-IL-1Ra and anti-PGRN antibodies were analyzed by FPLC. Representative chromatograms of IL-1Ra (**B**) and PGRN immune complexes (**C**) in seropositive patients' plasma compared to seronegative controls (**D**, **E**) are shown. (**F**, **G**) Fractions from gel filtration resembling peaks (P1-P3) according to respective chromatograms in **B** and **C** were subjected to native gradient PAGE and Western blot in comparison to non-separated seropositive patients' plasma. (**G**) Similarly, as control fractions from gel filtration of seronegative patients' plasma according to respective chromatograms in **D** and **E** were subjected to native gradient PAGE and Western blot in comparison to non-separated patients' plasma. (**H**) Side-by-side analysis of gel filtration separated anti-IL-1Ra (left panel) and PGRN immune complexes (right panel) compared to COVID-19 patients' plasma as in **Figure 1B** and **D** on native gradient PAGE followed by Western blot.

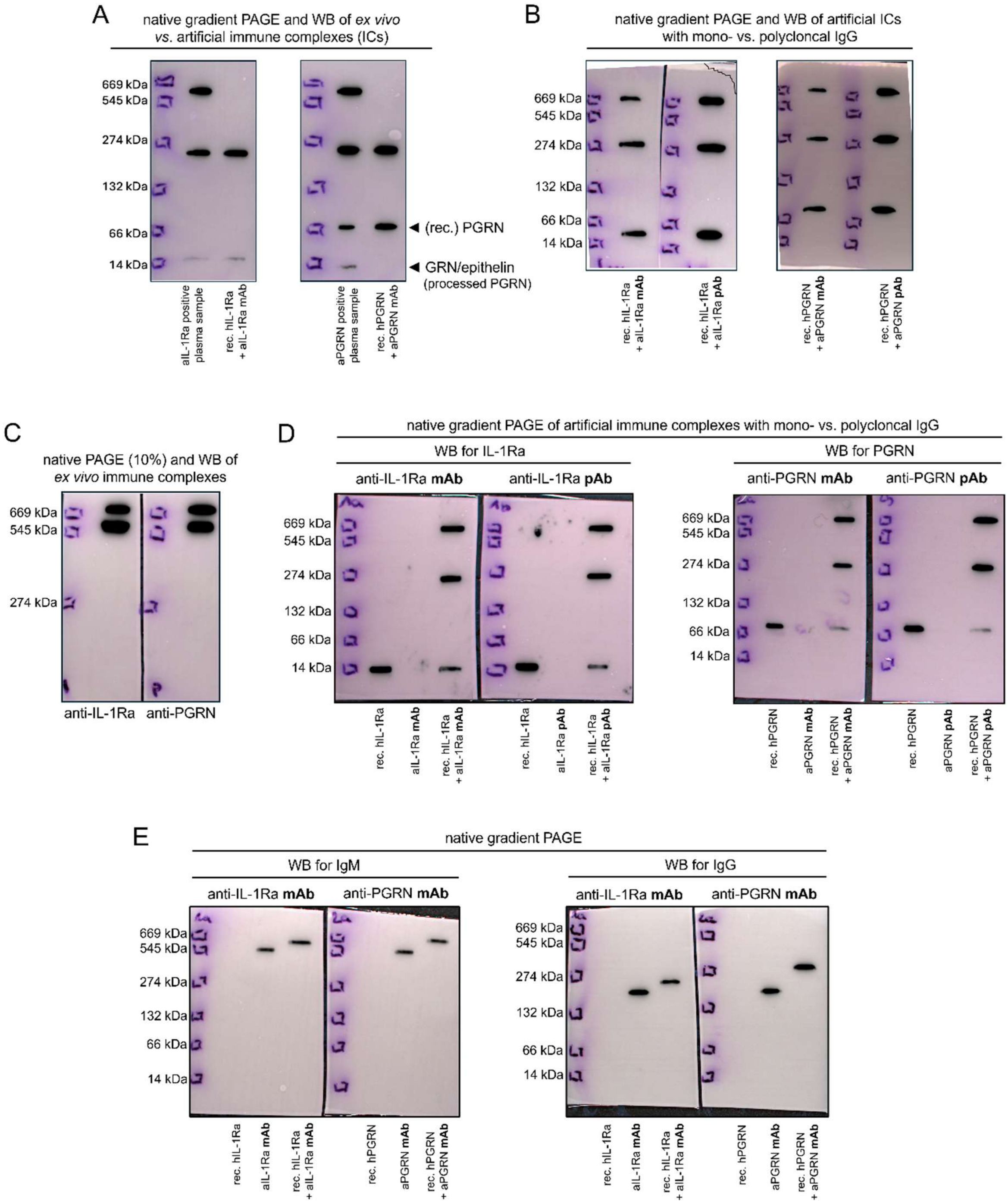

Supplementary Figure 8. Artificial IL-1Ra and PGRN immune complexes. (**A-E**) Artificial immune complexes from recombinant antigen and mono- or polyclonal commercial anti-IL-1Ra or anti-PGRN antibody preparations (mAb, pAb) were analyzed by native gradient PAGE and Western blot in comparison to patients' plasma (**A**). (**A**) Patients' plasma can contain both processed granulins (GRN, also termed epithelin, approx. 10kDa) as well as progranulin (PGRN, approx. 80kDa). (**C**) Patient's plasma analyzed for anti-IL-1Ra and anti-PGRN immune complexes on 10% native PAGE followed by Western blot. (**D**) Native gradient PAGE and Western blot (for IL-1Ra, left panels; for PGRN, right panels) of all components

(commercial anti-IL-1Ra and anti-PGRN mAbs, recombinant antigens) used to assemble artificial immune complexes. (**E**) Native gradient PAGE and Western blot (for IgM, left panels; for IgG, right panels) of all components (commercial anti-IL-1Ra and anti-PGRN mAbs, recombinant antigens) used to assemble artificial immune complexes.

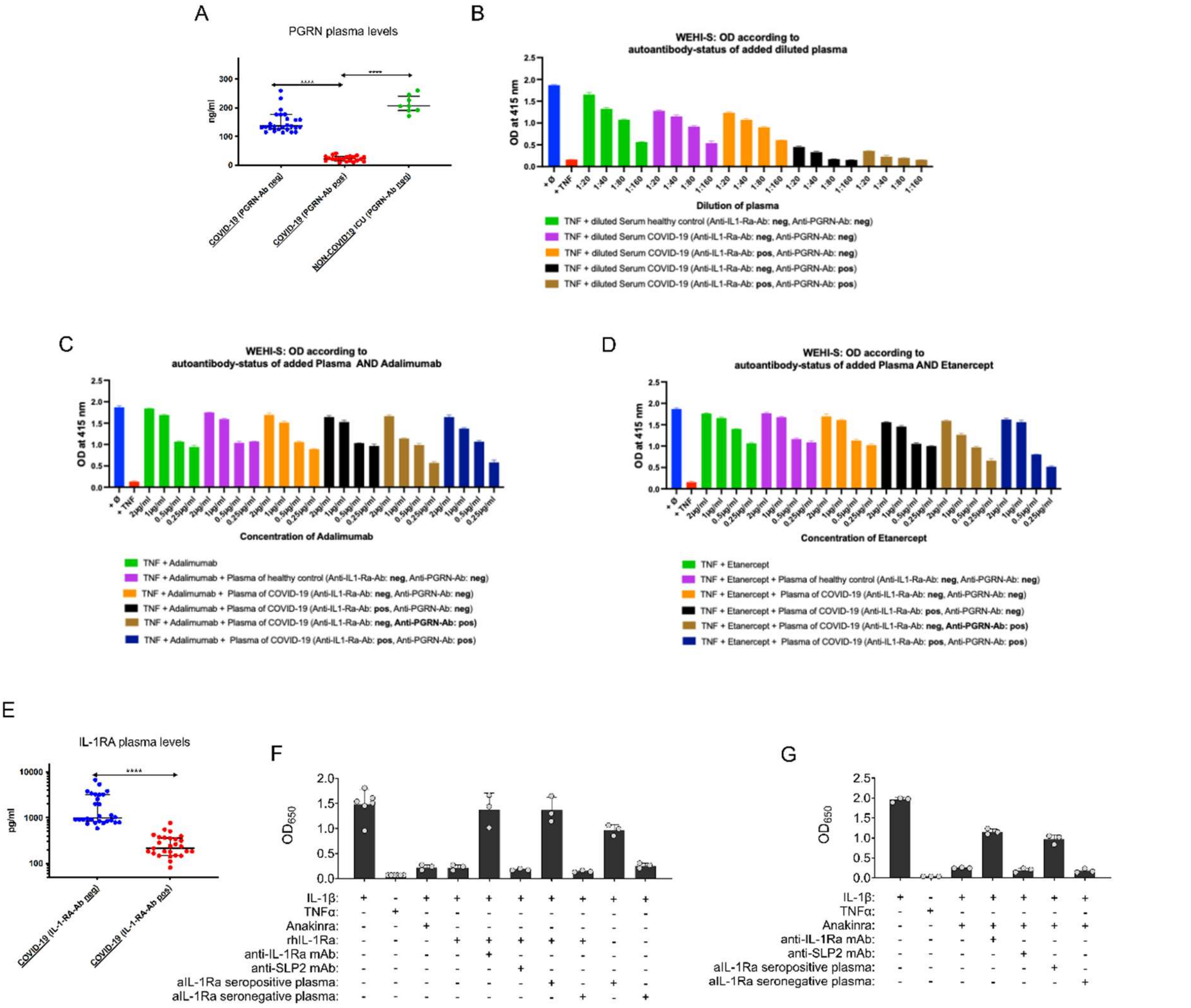

Supplementary Figure 9. Antibody-mediated depletion of peripheral PGRN and IL-1Ra and impairment of anti-inflammatory bioactivity. (**A**) PGRN plasma levels in anti-PGRN seropostive (n=21) or seronegative COVID19 patients (n=29) or non-COVID ICU controls (n=8) was determined by commercial ELISA. Lines in scattered dot plots indicate median and interquartile range. Data were analyzed by Kruskal-Wallis followed by Dunn's post-hoc test. (**B**) To test for impact of anti-PGRN antibodies on PGRN bioactivity WEHI-S cells were incubated with TNF and plasma from selected discovery cohort COVID19 patients at different dilutions. TNF-toxicity on WEHI-S cells was quantified by MTT assay. (**C**, **D**) Experiments as in (**B**) but including addition of Adalimumab (C) or Ethanercept (D) at indicated concentrations. (**E**) IL-1Ra plasma levels in anti-IL-1Ra seropostive (n=29) or seronegative COVID19 patients (n=28) was determined by respective commercial ELISA. Lines in scattered dot plots indicate median and interquartile range. Data were analyzed by Mann-Whitney U test. (**F**, **G**) To test for functional impact of anti-IL-1Ra antibodies HEK IL-1 reporter cells were treated with the indicated stimuli and agents and plasma samples. A recombinant anti-IL-1Ra and anti-SLP2 antibody served as positive and negative control, respectively. SEAP release by reporter cells upon effective IL-1 signaling was quantified at 650 nm. Data from two independent experiments with measurements in triplicates (or more) are shown.

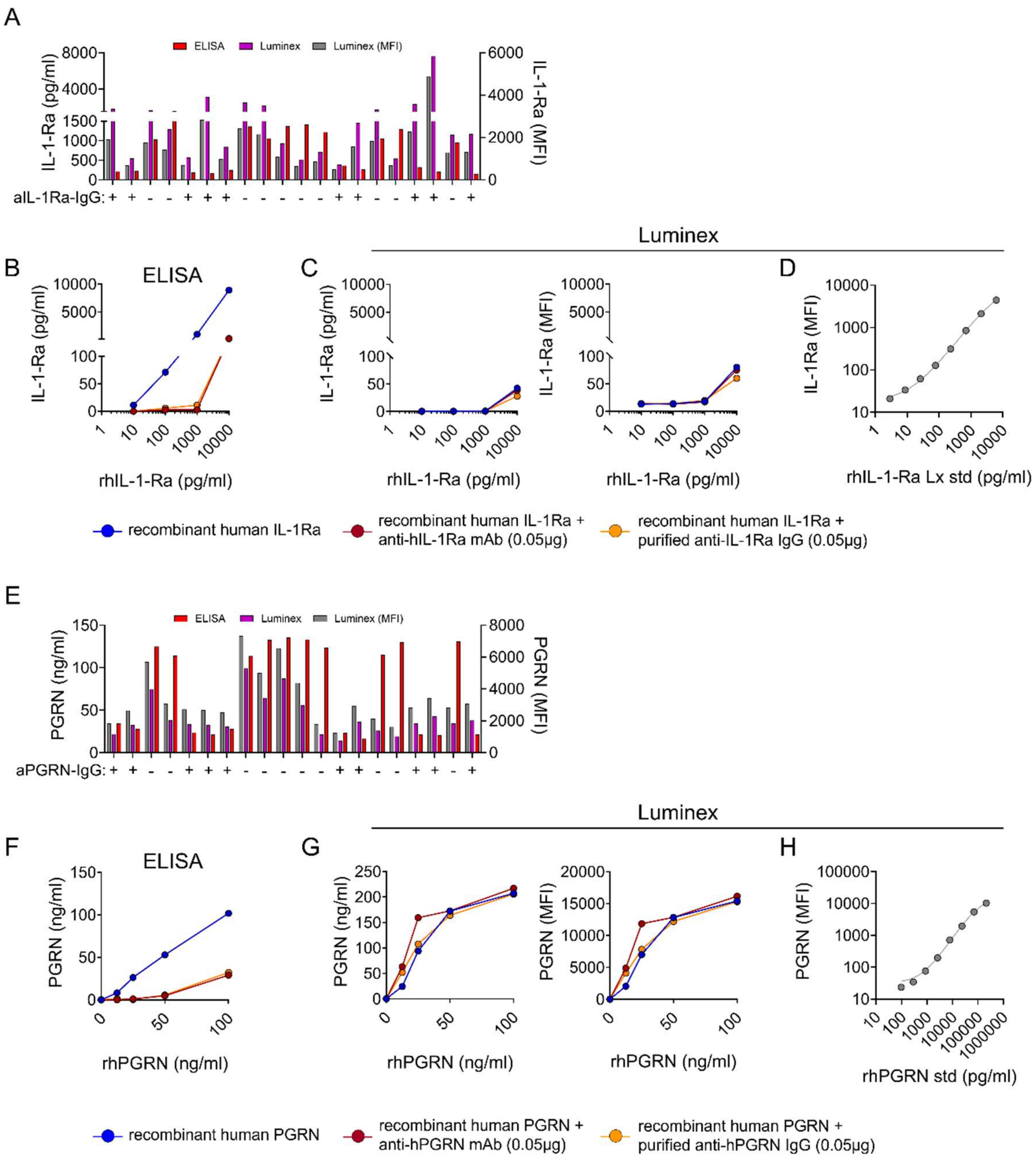

Supplementary Figure 10. PGRN and IL-1Ra detection in commercial ELISAs versus Luminex. (**A**) IL-1Ra plasma levels in validation cohort patients was determined by commercial ELISA and commercial Luminex reagents. IL-1Ra levels are given in absolute concentration (pg/mL) as well as raw mean fluorescence intensity (MFI, Luminex). Anti-IL-1Ra status as determined by ELISA is indicated on the x-axis. (**B**, **C**) Quantification of IL-1Ra by commercial ELISA (**B**) or Luminex (**C**) in samples spiked with recombinant IL-1Ra (0-10.000pg/mL) with or without addition of 0.05µg/mL of commercial anti-IL-1Ra mAb or anti-IL-1Ra IgG purified from patient's plasma (Val16). C) IL-1Ra standard curve on Luminex. (**E**) PGRN plasma levels in validation cohort patients as determined by commercial ELISA and commercial Luminex reagents. PGRN levels are given in absolute concentration (pg/mL) as well as raw mean fluorescence intensity (MFI, Luminex). Anti-PGRN status as determined by ELISA is indicated

on the x-axis. (**F**, **G**) Quantification of PGRN by commercial ELISA (**F**) or Luminex (**G**) in samples spiked with recombinant PGRN (0, 12.5, 25, 50 and 100pg/mL) with or without addition of 0.05µg/mL of commercial anti-PGRN mAb or anti-PGRN IgG purified from patient's plasma (Val16). (**H**) IL-1Ra standard curve on Luminex.

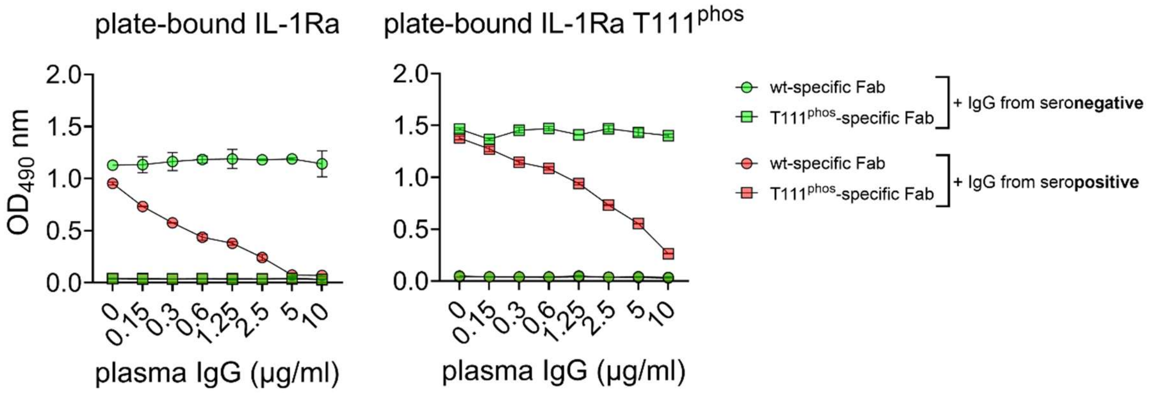

Supplementary Figure 11. Competition of selected Fab-fragments with patients' IgG for antigen binding. Fab fragments selected against canonically phosphorylated or hyperphosphorylated IL-1Ra ($T111^{phos}$) at fixed concentration (1µg/ml) were set up to compete for plate-bound antigen binding with indicated concentrations of IgG purified from anti-IL-1Ra seropostivie or seronegative plasma. Data depict mean and standard deviation of measurements performed in duplicates.

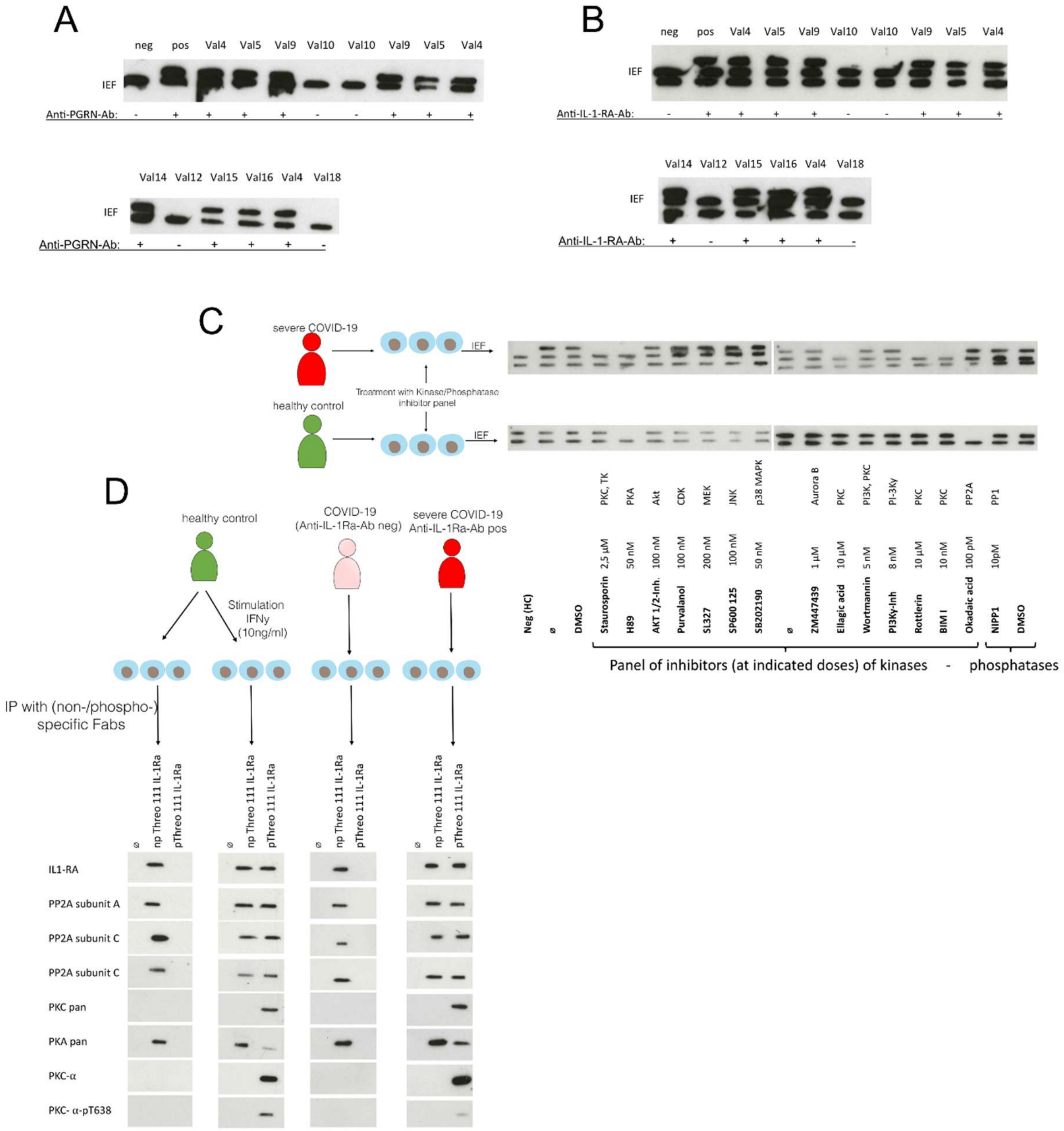

Supplementary Figure 12. Antigen hyperphosphorylation among validation cohort patients and of PGRN and involvement of monocytic kinases. (**A**, **B**) IEF of PGRN (**A**) and IL-1Ra (**B**) in validation cohort COVID19 plasma samples previously tested seropositive or seronegative for anti-PGRN or anti-IL-1Ra antibodies. (**C**) Monocytes from healthy control and one patient with severe COVID19 and seropositive for anti-IL-1Ra antibodies were treated with indicated kinase and phosphatase inhibitors. Impact on IL-1Ra phosphorylation was assessed by IEF of cell lysates. (**D**) Monocytes from healthy control with or without *ex vivo* stimulation with IFNγ (for 48h, 37°C), one patient with severe COVID19 and seropositive for anti-IL-1Ra antibodies and one seronegative COVID19 patient were lyzed and cell lysates were subjected to IP using T111- and T111^{phos}-specific Fabs. IP-eluates were analyzed by western blot by staining for indicated kinases and subunits.

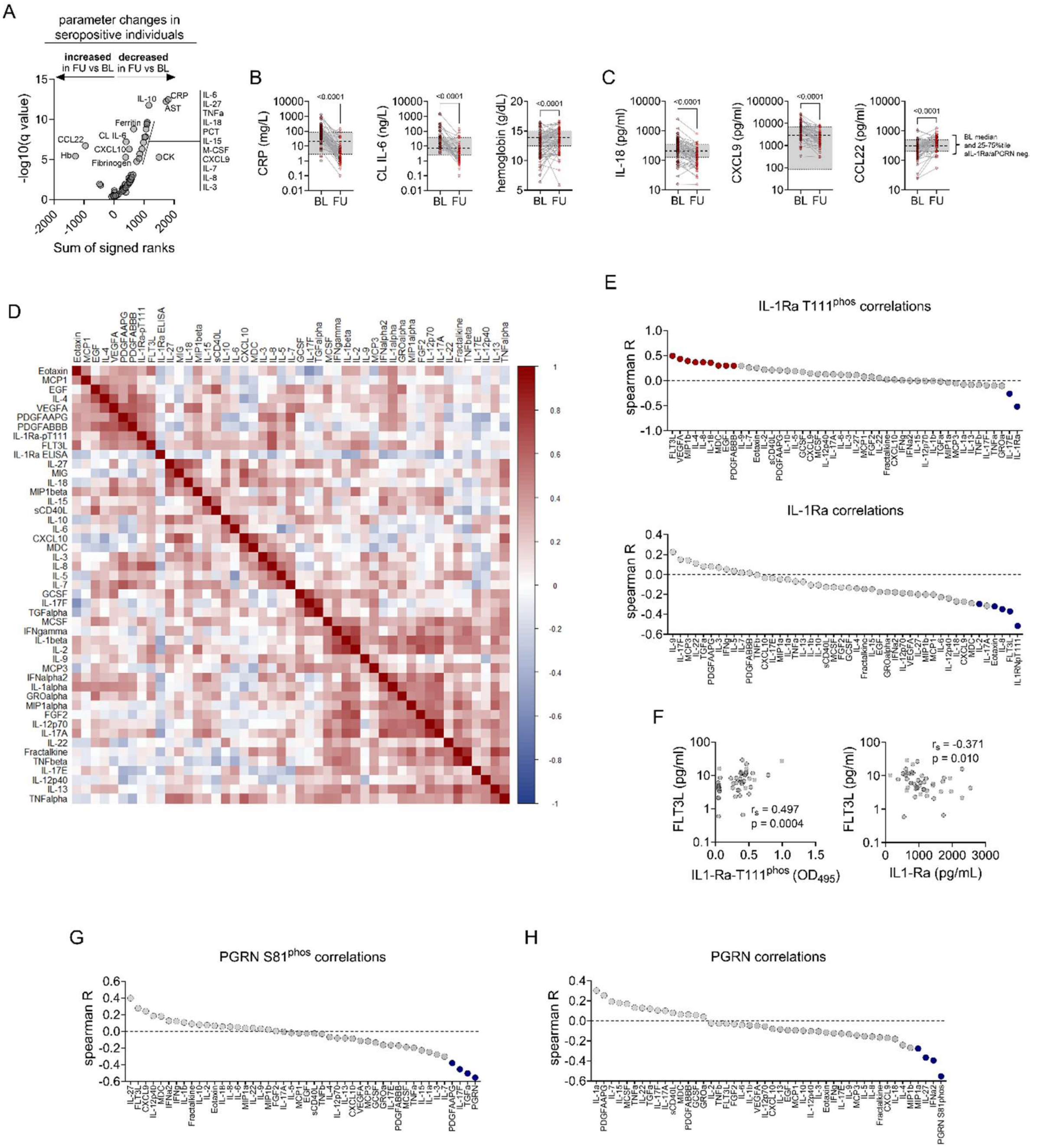

Supplementary Figure 13. (Hyper)inflammatory mediator signatures and association with autoantibody seropositivity and presence of hyperphosphorylated antigen in NAPKON samples in course of follow-up. (**A**) Inflammatory mediator changes from baseline to 3-month follow-up as determined by 45plex bead array. (**B**, **C**) Paired observations (where data were available) for clinical laboratory parameters (**B**; CRP: n=61; IL-6: n=36; hemoglobin: n=65) and markers from an experimental 45plex bead array assay (**C**; all n=48) with significantly different levels at baseline and 3-month follow-up in NAPKON patients with severe COVID19 at baseline and seropositive for both anti-IL-1Ra and anti-PGRN antibodies. Data were analyzed by were analyzed by two-tailed Wilcoxon signed rank test for paired non-parametric

observations. Dashed horizontal line and grey bar in each blot indicates median baseline levels and 25-75percentile of respective baseline levels in seronegative patients' plasma. (**D**) Multiple correlation analysis of inflammatory mediator levels determined by 45plex bead array assay and in-house ELISA (IL-1Ra, $T111^{phos}$ IL-1Ra) in seropositive severe COVID-19 NAPKON samples at 3-month follow-up. (**E**) Multiple association analysis of inflammatory mediator levels determined by 45plex bead array assay and in-house ELISA (IL-1Ra, $T111^{phos}$ IL-1Ra) with presence of hyperphosphorylated IL-1Ra (**E**, upper panel; $T111^{phos}$ IL-1Ra) and endogenous IL-1Ra levels (**E**, lower panel) in severe COVID-19 NAPKON 3-month follow-up samples. **(F)** Correlation of FLT3L plasma levels with presence of hyperphosphorylated IL-1Ra (**F**, upper panel; $T111^{phos}$ IL-1Ra; n=47) and endogenous IL-1Ra levels (**F**, lower panel; n=47) in severe COVID-19 NAPKON 3-month follow-up samples. (**G**) Multiple association analysis of inflammatory mediator levels determined by 45plex bead array assay and in-house ELISA (PGRN, $S81^{phos}$ PGRN) with presence of hyperphosphorylated PGRN (**G**, left panel; $S81^{phos}$ PGRN) and endogenous PGRN levels (**G**, right panel) in severe COVID-19 NAPKON 3-month follow-up samples.

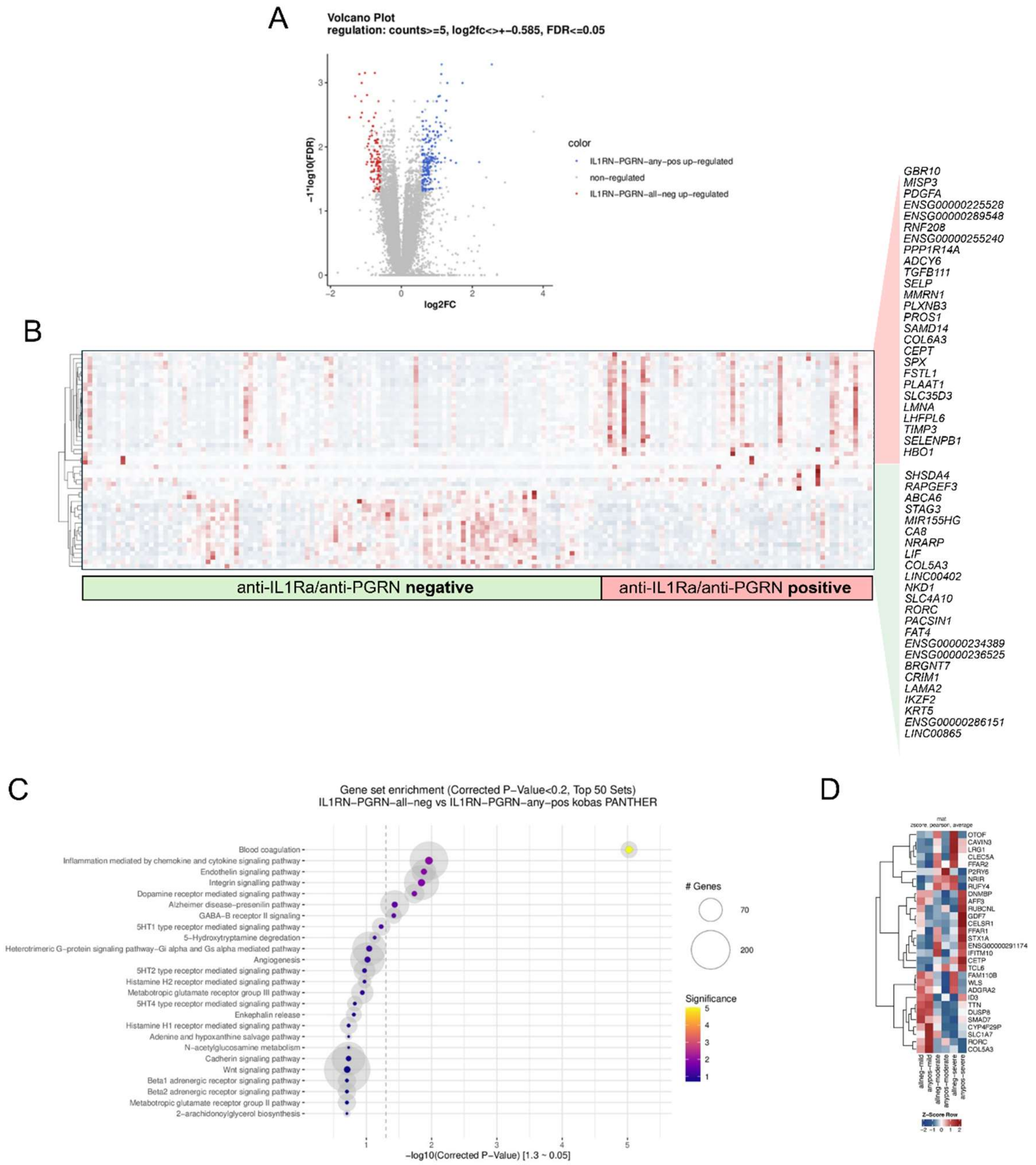

Supplementary Figure 14. RNA sequencing of whole blood cells from COVID-19 patients enrolled in NAPKON. (**A**) Volcano plot of differentially expressed genes. RNA expression compared of seropositive vs. seronegative patients of NAPKON validation cohort. (**B**) Top-50 differentially expressed genes. (**C**) Gene set enrichment analysis. (**D**) Differentially expressed genes when comparing RNA expression only of patients with servere COVID-19 seropositive vs. seronegative autoantibodies.

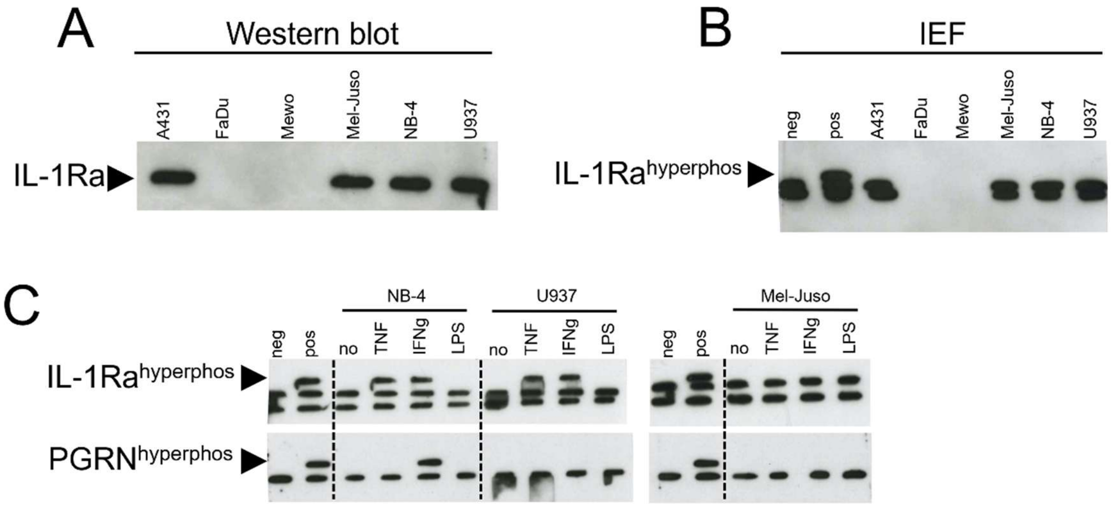

Supplementary Figure 15. PGRN and IL-1Ra (hyper)phosphorylation in different cells lines. (**A**, **B**) Western blot for IL-1Ra expression (**A**) and IEF for IL-1Ra (hyper)phosphorylation (**B**) in cell lysates of the indicated cell lines. (**C**) IEF of IL-1Ra and PGRN in cell lysates of NB4, U937 and Mel-Juso cell lines following stimulation for 48h at 37°C with recombinant TNF, IFNγ or LPS (10ng/ml.

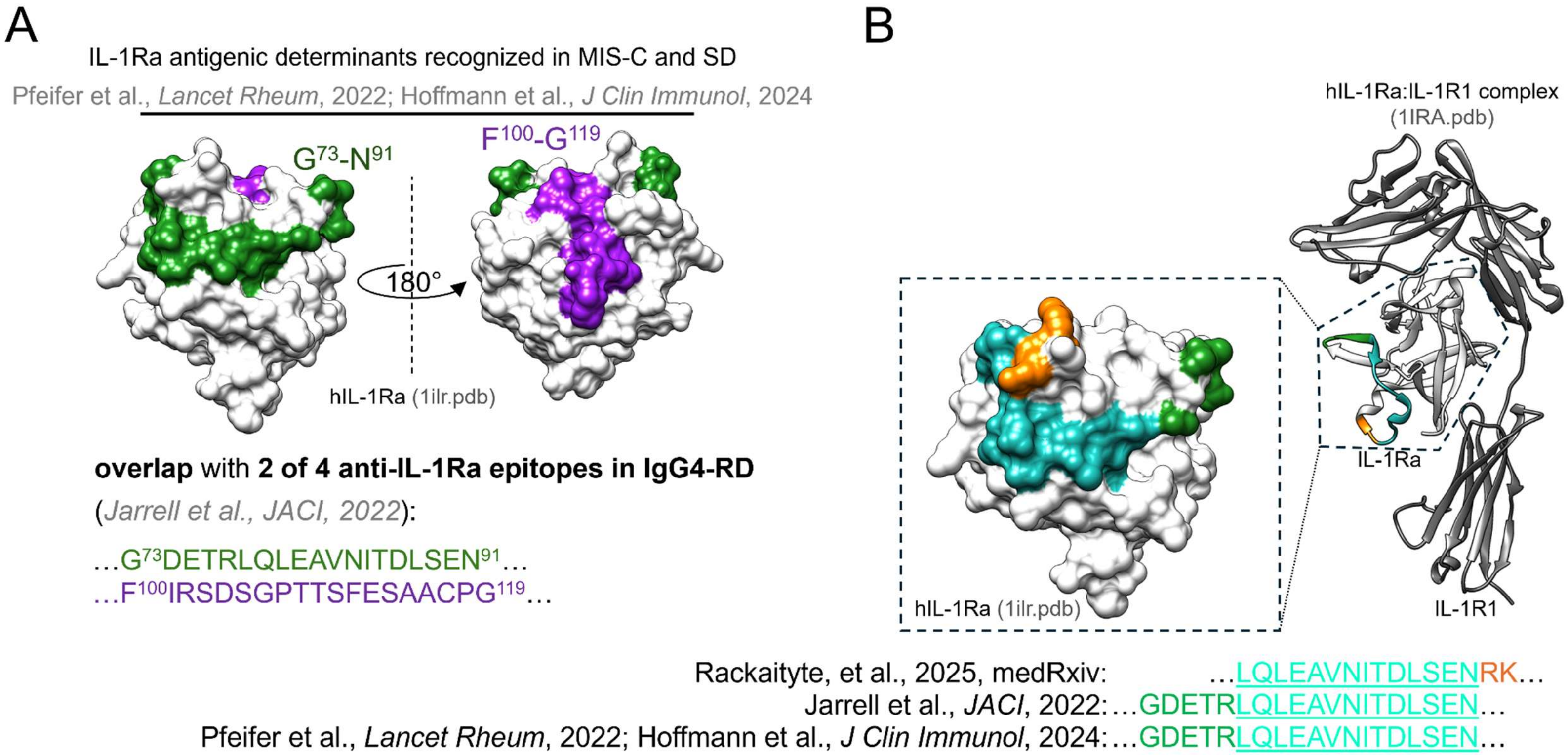

Supplementary Figure 16. Antigenic determinants recognized by anti-IL-1Ra autoantibodies. (**A**, **B**) Epitope mapping data reported with our previous studies (Pfeifer et al., 2022; Hoffmann et al., 2024) as well as others (Jarrell et al., 2022; Rackaityte et al., 2025, doi: 10.1101/2024.10.03.24314850) describing the occurrence of IL-1Ra-targeting autoantibodies in different clinical context. (**A**) Overlapping epitopes determined upon IgG-binding to recombinant IL-1Ra fragments in MIS-C or Still's Disease (SD) with those determined in context of IgG4 related disease (IgG4-RD) are visualized on a surface representation of human IL-1Ra (based on 1lir.pdb). (**B**) A common epitope recognized by anti-IL-1Ra antibodies determined in different studies is visualized on a surface representation of human IL-1Ra (based on 1lir.pdb) as well as a ribbon representation of IL-1Ra bound to IL-1 receptor 1 (IL-1R1; based on 1IRA.pdb). Figure S16 is based on a reply letter figure originally included with the R1 revision of the present study.

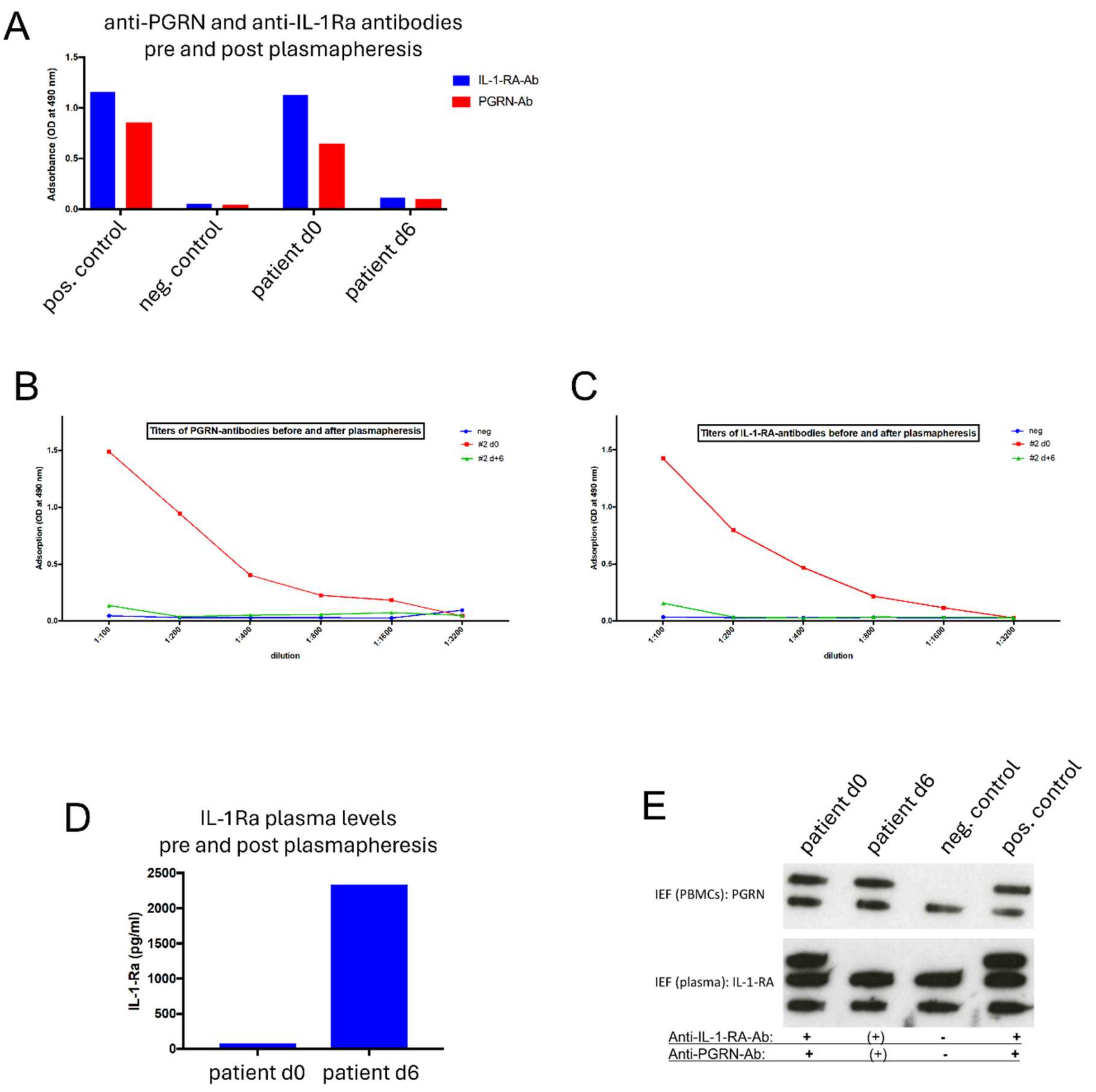

Supplementary Figure 17. Anti-PGRN and anti-IL-1Ra antibodies in a COVID19 patient in course of plasmapheresis. (**A**) Presence of anti-PGRN and anti-IL-1Ra antibodies in plasma of discovery cohort COVID19 patient #2 before (d0) and after a cycle of plasmapheresis (d6) were determined by respective in-house ELISA. (**B**, **C**) Anti-PGRN and anti-IL-1Ra antibody titers in plasma of discovery cohort COVID19 patient #2 before (d0) and after a cycle of plasmapheresis (d6) were determined by serial dilution of plasma and respective in-house ELISA. (**D**) IL-1Ra plasma levels before (d0) and after plasmapheresis (d6) were assessed by commercial ELISA. (**E**) IEF of PGRN and IL-1Ra in PBMC cell lysates before (d0) and after plasmapheresis (d6).
