# Supplementary material for: Autoantibodies to IL-1Ra and PGRN in severe COVID-19 are associated with inflammation-induced hyperphosphorylated antigen isoforms
Source: Nat Commun. 2026 May 27;17:4768. doi: 10.1038/s41467-026-73316-5 (PMC13219524; doi:10.1038/s41467-026-73316-5)
Supplement: Supplementary file 2 — Description of Additional Supplementary Files [file 41467_2026_73316_MOESM2_ESM.pdf]

Description of Additional Supplementary Files

Supplementary Data 1: COVID19 discovery cohort

Supplementary Data 2: non-COVID-19 ICU controls

Supplementary Data 3: COVID-19 validation cohort 1

Supplementary Data 4: COVID19 validation cohort 2 (NAPKON)
