# Supplementary material for: Autoantibodies to IL-1Ra and PGRN in severe COVID-19 are associated with inflammation-induced hyperphosphorylated antigen isoforms
Source: Nat Commun. 2026 May 27;17:4768. doi: 10.1038/s41467-026-73316-5 (PMC13219524; doi:10.1038/s41467-026-73316-5)
Supplement: Supplementary file 7 — Reporting Summary [file 41467_2026_73316_MOESM7_ESM.pdf]

nature portfolio

Corresponding author(s): Lorenz Thurner

Last updated by author(s): 2026 04 16

Reporting Summary

Nature Portfolio wishes to improve the reproducibility of the work that we publish. This form provides structure for consistency and transparency in reporting. For further information on Nature Portfolio policies, see our Editorial Policies and the Editorial Policy Checklist.

Statistics

For all statistical analyses, confirm that the following items are present in the figure legend, table legend, main text, or Methods section.

n/a	Confirmed	
☐	☒	The exact sample size (*n*) for each experimental group/condition, given as a discrete number and unit of measurement
☐	☒	A statement on whether measurements were taken from distinct samples or whether the same sample was measured repeatedly
☐	☒	The statistical test(s) used AND whether they are one- or two-sided<br>*Only common tests should be described solely by name; describe more complex techniques in the Methods section.*
☐	☒	A description of all covariates tested
☐	☒	A description of any assumptions or corrections, such as tests of normality and adjustment for multiple comparisons
☐	☒	A full description of the statistical parameters including central tendency (e.g. means) or other basic estimates (e.g. regression coefficient) AND variation (e.g. standard deviation) or associated estimates of uncertainty (e.g. confidence intervals)
☐	☒	For null hypothesis testing, the test statistic (e.g. *F*, *t*, *r*) with confidence intervals, effect sizes, degrees of freedom and *P* value noted<br>*Give P values as exact values whenever suitable.*
☒	☐	For Bayesian analysis, information on the choice of priors and Markov chain Monte Carlo settings
☒	☐	For hierarchical and complex designs, identification of the appropriate level for tests and full reporting of outcomes
☐	☒	Estimates of effect sizes (e.g. Cohen's *d*, Pearson's *r*), indicating how they were calculated

Our web collection on statistics for biologists contains articles on many of the points above.

Software and code

Policy information about availability of computer code

Data collection	Microplate Manager 6 version 6.3; EvolutionCapt edge x64; Luminex xPONENT version 4.2.; Trimmomatic version 0.39; STAR 2.7.11b; Picard 3.1.1; featureCounts 2.0.4; DESeq2 version 1.36.0
Data analysis	GraphPad Prism version 8.0 for Mac OS X and version 10 for windows, Graphpad Software, La Jolla, CA, USA<br><br>Following tests were used: Classical statistics (GraphPad / cohort comparisons)<br><br>• D’Agostino & Pearson normality test<br>• Mann–Whitney test (Mann–Whitney U test)<br>• Kruskal–Wallis test<br>• Dunn’s multiple comparison test / Dunn’s post-hoc test<br>• Friedman test (multiple paired observations)<br>• Wilcoxon test (paired non-parametric observations)<br>• Fisher’s exact test (two-tailed)<br>• Spearman rank correlation<br><br>RNA-seq / pathway & enrichment statistics<br>• Wald test (DESeq2 differential expression p-values)<br>• Hypergeometric test (KOBAS gene set overrepresentation)<br>• Multi-level split Monte-Carlo scheme (fGSEA p-values)

• z-test (Pscan TFBS p-values)
• Two-component Gaussian mixture model fit (TOBIAS; to set bound/unbound threshold)

Multiple-testing / p-value handling explicitly stated
• Benjamini–Hochberg correction / method (used repeatedly; e.g., BH-corrected p-values, and noted as correction for multiple testing)
• Uncorrected p-value thresholding (e.g., TFBS enrichment shown with uncorrected p-value < 0.05)
• "No multiple-testing correction implemented/available by default" (Pscan / TOBIAS statements)
for RNA seq analysis of NAPKON samples please see statistic in Methods.

R-studio for generating colleration matrices incl. hierachical clustering using the corrplot R package and Rstudio (RStudio Team (2015). RStudio: Integrated Development for R. RStudio, Inc., Boston, MA http://www.rstudio.com/).

For manuscripts utilizing custom algorithms or software that are central to the research but not yet described in published literature, software must be made available to editors and reviewers. We strongly encourage code deposition in a community repository (e.g. GitHub). See the Nature Portfolio guidelines for submitting code & software for further information.

Data

Policy information about availability of data

All manuscripts must include a data availability statement. This statement should provide the following information, where applicable:

- Accession codes, unique identifiers, or web links for publicly available datasets
- A description of any restrictions on data availability
- For clinical datasets or third party data, please ensure that the statement adheres to our policy

All data required to evaluate the conclusions in the paper are present in the manuscript or its appendix as well as the source data file. Our study reports on proteomic and whole blood RNA sequencing data that were generated by the NAPKON consortium. Researchers can access all NAPKON data through the UAC procedure (https://napkon.de/use-and-access/). The project underlying this publication was granted access to the data set from the NAPKON UAC under the ID 2022-08-17.

Research involving human participants, their data, or biological material

Policy information about studies with human participants or human data. See also policy information about sex, gender (identity/presentation), and sexual orientation and race, ethnicity and racism.

Reporting on sex and gender	This is reported with clinical characteristics in case this information was available to us
Reporting on race, ethnicity, or other socially relevant groupings	Such information was not available to us and no such information was reported
Population characteristics	reported in Methods and results
Recruitment	Plasma samples of discovery, validation and control cohorts were locally obtained from the CORSAAR registry study (Saarland, Germany). From few patients enrolled in CORSAAR we also prepared PBMCs. All patients or their legal representatives provided written informed consent. Analysis for proinflammatory autoantibodies and in-depth characterization in samples of CORSAAR and other trials was also approved by the local Ethical Review Board (Ethics Committee of Saarland University, Homburg, Germany; Bu42/21). These other trials/registries comprised plasma samples obtained from convalescent individuals enrolled in the CAPSID trial (EUDRA-CT 2020-001310-38), samples of the CoKiBa study (research project of COVID-19 in children and adolescents by a network of local practicing pediatricians and the University Hospital Regensburg obtained 1-3 months after the peak of the first pandemic wave in spring 2020 in Bavaria, Germany), samples of patients with severe, moderate and mild COVID-19 enrolled in NAPKON (National Pandemic Cohort Network, Germany), and pre-pandemic infectious control samples of the MAXSEP (NCT00534287) and the SISPCT trial (NCT00832039) of the German Sepsis Network. Please see Supplementary Fig. 1 for numbers (n) and the use of these samples in the context of the present study. All participants or their legal representatives provided written informed consent to the collection of blood samples and clinical data as part of these studies or registries.
Ethics oversight	Ethics Committee of Saarland University, Homburg, Germany; Bu 62/20 and Bu42/21

Note that full information on the approval of the study protocol must also be provided in the manuscript.

Field-specific reporting

Please select the one below that is the best fit for your research. If you are not sure, read the appropriate sections before making your selection.

☒ Life sciences ☐ Behavioural & social sciences ☐ Ecological, evolutionary & environmental sciences

For a reference copy of the document with all sections, see nature.com/documents/nr-reporting-summary-flat.pdf

Life sciences study design

All studies must disclose on these points even when the disclosure is negative.

Sample size	The study was conceptualized and performed already in 2021. Sample size was largely driven by sample availability and access to stated registries and/or study groups.
Data exclusions	samples with other diseases as initially reported.
Replication	Many experiments are screeing experiments, where several methods (ELISA, PAGE, IEF and western blots) were performed on single patient samples in order to validate results using different methodology. For some experiments, those were repeated on biomaterial from several individual donors at least twice. Data for such replications are shown in the manuscript or supplement and provided in the source data file.
Randomization	n/a as no randomization, no interventional trial
Blinding	Analysis of NAPKON samples for autoantibodies was performed without knowledge of the underlying clinical severity. Luminex on patient plasma as well as whole blood RNA sequencing was performed blinded for autoantibody status.

Reporting for specific materials, systems and methods

We require information from authors about some types of materials, experimental systems and methods used in many studies. Here, indicate whether each material, system or method listed is relevant to your study. If you are not sure if a list item applies to your research, read the appropriate section before selecting a response.

Materials & experimental systems

n/a	Involved in the study	
☐	☒	Antibodies
☐	☒	Eukaryotic cell lines
☒	☐	Palaeontology and archaeology
☒	☐	Animals and other organisms
☒	☐	Clinical data
☒	☐	Dual use research of concern
☒	☐	Plants

Methods

n/a	Involved in the study	
☒	☐	ChIP-seq
☒	☐	Flow cytometry
☒	☐	MRI-based neuroimaging

Antibodies

Antibodies used

Use of all listed antibodies is detailed in the methods section
murine anti-FLAG mAb, 1:2500; Sigma-Aldrich, Munich, Germany, Prod. No. F3165
biotinylated goat anti-human heavy and light chain IgG, 1:2500; Dianova, Hamburg, Germany, Prod. No. DNA-SEC183078
sheep anti-human IgG1, 1:5000; Binding Site Group, Birmingham, UK, Prod. Nos. AU006
sheep anti-human IgG2, 1:5000; Binding Site Group, Birmingham, UK, Prod. Nos. AU007
sheep anti-human IgG3, 1:5000; Binding Site Group, Birmingham, UK, Prod. Nos. AU008
sheep anti-human IgG4, 1:5000; Binding Site Group, Birmingham, UK, Prod. Nos. AU009
goat anti-human IgM, 1:2500; Dianova, Prod. No. DNA-SEC183006)
goat anti-human IgA, 1:2500; Dianova, Prod. No. DNA-SEC183007
anti-sheep IgG-POX, 1:5000; Sigma Aldrich, Prod. No. A9452
biotinylated anti-goat IgG, 1:2500; Dianova, Prod. No. DNA-SEC182876
murine anti-hPGRN antibody, 5 µg/mL or 1:2000, depending on experiment; abcam Prod. No. ab169325
biotinylated goat anti-human IgG (F(ab')2 antibody, 1:2500; Dianova, Prod. No. 109-066-097
rabbit anti-human IL-1Ra antibody, 1 or 5 µg/mL or 1:2000, depending on experiment; antibodies-online, Prod. No. ABIN2856394
murine anti-human PGRN polyclonal Ab, 1µg/mL; antibodies online Prod. No. ABIN11169131
recombinant SLP-antibody, 5µg/mL; abcam, Prod. No. ab191883
rabbit anti-FLAG antibiody, 1:2500; Sigma Aldrich, Prod. No. F7425
murine anti-human PGRN antobody, 1µg/mL or concentration range as indicated in Supplementary figure 5C-F; antibodies online, Prod. No. ABIN516191
murine anti-human IL-1Ra mAb, 1µg/mL or concentration range as indicated in Supplementary figure 5C-F; antibodies online, Prod. No. ABIN7425773
biotinylated goat anti-murine IgG, 1:2500; Biozol, Prod. No. DNA-SEQ-183154
mouse-anti- HIS antibody, 1:2000; Qiagen, Prod. No. 34660
biotinylated anti-mouse IgG antibody, 1:2500; Dianova, Prod. No. DNA-SEC183803

Validation

Phage-display selected Fabs with specificity for IL-1RaT111phos were assessed for respective specificity with IEF

Eukaryotic cell lines

Policy information about cell lines and Sex and Gender in Research

Cell line source(s)	HEK293 (in house)<br>A431 (DSMZ, Braunschweig, Germany, Prod. No. ACC91)<br>FaDu (DSMZ, Prod. No. ACC784)<br>Mewo (ATCC, Prod. No. HTB-65)<br>Mel-Juso (DSMZ, Prod. No. ACC74)<br>NB4 (DSMZ, Prod. No. ACC207)<br>U937 (DSMZ, Prod. No. ACC5)<br>WEHI-S cell line (DSMZ, Prod. No. ACC25)<br>HEK-Blue™ IL-1β reporter cells (Invivogen, Prod. No. hkb-il1bv2)
Authentication	Cell lines used in our study were not independently authenticated by us
Mycoplasma contamination	Mycoplasma contamination was excluded by performing regular PCR-testing
Commonly misidentified lines (See ICLAC register)	n.a.

Plants

Seed stocks	*Report on the source of all seed stocks or other plant material used. If applicable, state the seed stock centre and catalogue number. If plant specimens were collected from the field, describe the collection location, date and sampling procedures.*
Novel plant genotypes	*Describe the methods by which all novel plant genotypes were produced. This includes those generated by transgenic approaches, gene editing, chemical/radiation-based mutagenesis and hybridization. For transgenic lines, describe the transformation method, the number of independent lines analyzed and the generation upon which experiments were performed. For gene-edited lines, describe the editor used, the endogenous sequence targeted for editing, the targeting guide RNA sequence (if applicable) and how the editor was applied.*
Authentication	*Describe any authentication procedures for each seed stock used or novel genotype generated. Describe any experiments used to assess the effect of a mutation and, where applicable, how potential secondary effects (e.g. second site T-DNA insertions, mosiacism, off-target gene editing) were examined.*
